# Organization of the stalk system on electrocytes in mormyrid weakly electric fish *Campylomormyrus compressirostris*

**DOI:** 10.1007/s00441-024-03938-y

**Published:** 2024-12-20

**Authors:** Otto Baumann, Feng Cheng, Frank Kirschbaum, Ralph Tiedemann

**Affiliations:** 1 Unit of Animal Physiology, Institute of Biochemistry and Biology, University of Potsdam, 14476 Potsdam, Germany; 2 Unit of Evolutionary Biology/Systematic Zoology, Institute of Biochemistry and Biology, University of Potsdam, 14476 Potsdam, Germany

**Keywords:** Electrocyte, Sodium pump, Plasma membrane Ca^2+^-ATPase, Confocal microscopy, F-actin

## Abstract

The adult electric organ in weakly electric mormyrid fish consists of action-potential-generating electrocytes, structurally and functionally modified skeletal muscle cells. The electrocytes have a disc-shaped portion and, on one of its sides, numerous thin processes, termed stalklets. These unite to stalks leading to a single main stalk that carries the innervation site. Here, we describe the 3-dimensional layout of the stalklet/stalk system in adult *Campylomormyrus compressirostris* by differential interference contrast microscopy and confocal fluorescence microscopy. Using antibodies against Na^+^/K^+^-ATPase α-subunit and plasma membrane Ca^2+^-ATPase, we show that these ion pumps are differentially distributed over the stalklet/stalk system, with plasma membrane Ca^2+^-ATPase being enriched on the stalklet membrane. Stalklets are distributed and organized in a quite uniform pattern on the posterior face of the electrocyte disc and fuse to terminal stalks. The latter then unite in a mostly dichotomic mode to stalks of increasing thickness, with the main stalk measuring about 100 µm in diameter. We further analyse the structural organization of stalklets and stalks, with a characteristic cytoskeletal system of bundled actin filaments in the centre and nuclei in subsurface position. These results suggest that the stalklet/stalk system is adapted in its structural layout to generate an action potential highly synchronized over the entire disc-portion of the electrocyte, accounting for the short electric organ discharge in this species. Our results suggest that actin-related proteins overexpressed in electrocytes, as shown previously by transcriptome analysis, may be involved in the organization of the unique F-actin system in stalklets and stalks.

**Supplementary Information:**

The online version contains supplementary material available at 10.1007/s00441-024-03938-y.

## Introduction

Electric organs (EOs) are composed of electrocytes, specialized cells that generate action potentials in a highly coordinated manner. The sum of these action potentials, an electric pulse called the electric organ discharge (EOD), produces an electric field in the surrounding media that is sensed by electroreceptor organs and can be used for location, social communication, prey catch and defence against predators (Crampton 2019; Caputi 2023). EOs evolved at least 6 times independently within vertebrates (Bass 1986a; Gallant 2019; Kirschbaum 2019; Wang and Yang 2021). Among these are the Mormyroidea, a superfamily of weakly electric fish that comprises 21 genera with more than 200 species endemic to Africa (Alves-Gomes and Hopkins 1997; Sullivan et al. 2000; Caputi 2023).

**Fig. 1:**
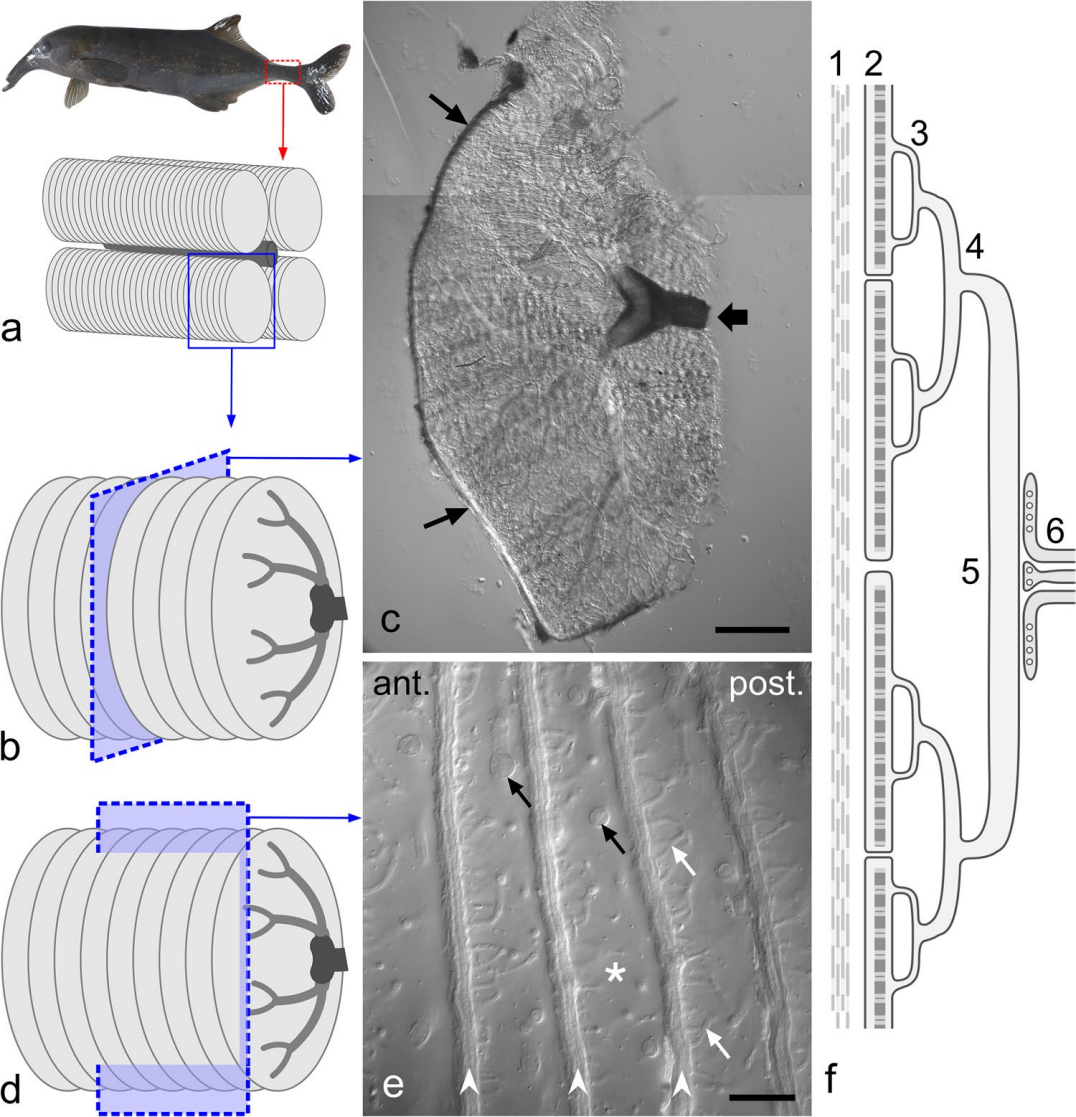
Organization of the adult electric organ (EO) in the mormyrid *C. compressirostris*. **a** The EO is located in the caudal peduncle (red box). Two columns of electrocytes are arranged on each side of the spine (dark grey structure in the centre). **b, c** Transverse planes through one of the 4 columns of electrocytes, shown in a schematic mode (**b**) and a DIC image of a ~ 200-µm-thick vibratome section (**c**). The electrocyte is seen *en face* view, with dense connective tissue on the outer surface (thin arrows) and the nerve approaching from the right (broad arrow). **d, e** Sagittal planes through an EO in a schematic view (**d**) and a DIC image (**e**), with anterior and posterior sides indicated. Electrocytes are stacked at a distance of about 175 µm within the EO column. On the posterior surface of the disc portion (arrowheads) of the electrocyte, thin stalklets emanate (white arrows). Stalklets and thicker stalks located further caudally (black arrows) reside in an extended extracellular space (asterisk). **f** Electrocyte presented in a schematic mode. A dense layer of collagen fibres (#1) resides on the anterior side of the electrocyte. Numerous stalklets (#3) extend from the posterior side of the main body (#2), fuse to stalks (#4) and finally to a main stalk (#5). The latter is innervated by a single nerve composed of numerous nerve fibres (#6). Scale bars: 500 µm (**c**), 100 µm (**e**)

The adult electric organ in mormyrid species is ontogenetically derived from myogenic tissue and located in the caudal peduncle (Szabo 1960; Denizot et al. 1982; Kirschbaum 1983; Gallant 2019). On each side of the spinal cord, flattened disc-shaped electrocytes are stacked behind each other in two columns that are ensheathed by insulating connective tissue (Fig. 1). These electrocytes are highly polarized in structural and functional aspects, with a rostral/anterior face and caudal/posterior face (Bass 1986a, b; Hopkins 1999; Markham 2013). Numerous thin processes termed stalklets protrude from one side of the electrocyte disc, in most species from the posterior side. Several of these stalklets then fuse to stalks, these unite more and more into stalks of increasing thickness, and ultimately to a single or a few main stalks innervated by electromotoneurons located in the spinal cord (Bruns 1971; Denizot et al. 1982). As the main stalk becomes depolarized in response to synaptic activity, the depolarization propagates along the stalk and stalklet system towards the electrocyte disc, causing a spike depolarization on its stalklet-carrying side and, after a short delay, a second spike on the opposite cell side (Stoddard and Markham 2008). As these two electrical discharges of an electrocyte are associated with ion currents directed in opposite directions with respect to the animal’s long axis, they create an extracellular biphasic electrical response, the micro-EOD of an electrocyte (Stoddard and Markham 2008; Markham 2019). The micro-EODs generated by all electrocytes within an EO are highly synchronized and sum up to the externally measurable EOD.

EODs generated by mormyrids are extremely diverse, species-specific (Bass 1986a, b; Hopkins 1999; Markham 2019), often sexually dimorphic (Bass et al. 1986), and may change during ontogeny (Denizot et al. 1982; Paul et al. 2015; Kirschbaum et al. 2016; Nguyen et al. 2020). EODs may differ by their polarity, signal waveform and especially in duration that varies from 0.2 ms to tens of ms (Hopkins 1999; Markham 2019). Several different factors may account for the enormous diversity of EODs in mormyrids. (1) EOs differ in the positioning of stalklets on the electrocyte main body, residing either on the posterior or the anterior face, and in whether the stalks remain on the same cell side or penetrate the disc once or even twice. These anatomical features can be correlated with the number and polarity of phases in the EOD (Szabo 1958; Bass 1986a; Alves-Gomes and Hopkins 1997; Gallant et al. 2011). (2) Surface proliferations on the main body and the density of ion channels within the membrane may influence membrane capacitance and resistance, respectively, and thus may affect the duration of EOD signals (Paul et al. 2015; Markham 2019; Korniienko et al. 2021). (3) EOs in different species may express different types of ion channels, with different voltage dependence and kinetics (Markham 2019). Notably, several studies have reported a differential expression for various K^+^ channel genes in correlation with the EOD duration, suggesting that these ion channels are potentially involved in EOD diversification (Nagel et al. 2017; Losilla et al. 2020; Cheng et al. 2024). (4) Paralogs for voltage-dependent ion channels with selective expression in the EO may have undergone rapid sequence evolution, leading to functional diversification of a distinct channel and EOD variation. This has been demonstrated to be the case for at least voltage-gated Na^+^ channel Na_v_1.4a (Zakon et al. 2006; Arnegard et al. 2010), voltage-gated K^+^ channel K_v_1.7a (Swapna et al. 2018) and inward-rectifying K^+^ channel Kir2.1 (Cheng et al. 2024). (5) The anterior and the posterior face of the main body may have a different repertoire of ion channels or a different density of these (Gallant et al. 2012; Markham 2019). This heterogeneity in molecular composition between both membrane areas may influence the temporal delay between the spikes on both cell faces and the shape of these spikes. (6) Finally, it has been suggested that structural features of the stalk system, such as stalk thickness and stalklet number, may affect temporal EOD parameters (Paul et al. 2015).

Whereas the geometry of electrocytes, the surface structure of the main body and the molecular differences between EOs (above #1 to 4) have attracted particular attention during the last years, the organization of the stalk system and its possible effect on the EOD (#6) has been largely neglected so far. Structural studies on mormyrid EOs to date have applied histological techniques in conjunction with either bright field microscopy or transmission electron microscopy, both providing only an extremely limited view on the 3-dimensional organization of the structurally complex stalklet/stalk system (Denizot et al. 1982; Bass et al. 1986; Paul et al. 2015; Nguyen et al. 2020; Korniienko et al. 2021). Actually, we suppose that not only structural features of the stalklet/stalk system are of importance for the EOD, but that the various segments of the stalklet/stalk system may also differ by molecular and functional properties, with possible impact on EOD generation and shape. Ideally, therefore, both the 3-dimensional organization of the stalklet/stalk system and the distribution of ion transporters and channels on it should be examined and compared between mormyrid species with different EODs.

The present study on *Campylomormyrus compressirostris* represents a starting point in this comprehensive endeavor. The EO in this species is characterised by its relative simple geometry, which is of the posterior non-penetrating type, with stalklets emanating on the posterior side and the stalks not penetrating the cell body (Fig. 1; Paul et al. 2015). The EOD is extremely short at less than 0.2 ms, biphasic, and without obvious changes during ontogeny (Paul et al. 2015; Kirschbaum et al. 2016). As pointed out by Bennett (1971), precondition for such a short EOD is a temporally synchronized propagation of depolarizations along the stalk arborisations in order to excite the cell body simultaneously over its entire posterior face. The (almost) complete genome of *C. compressirostris* has recently been published (Cheng et al. 2023), and differential gene expression analysis has provided information about the genes up- or downregulated in the EO in comparison to skeletal muscle in the same species, or to EOs in other *Campylomormyrus* species that have a comparatively long EOD (Cheng et al. 2024). We suppose that at least some of these gene products overexpressed in EOs are associated with the organization and functions of the cell structure which is specific for electrocytes, the stalklet/stalk system. Here we examine the 3D organization of the stalklet/stalk system by using differential interference contrast microscopy and confocal fluorescence microscopy, paying particular attention to features that could possibly contribute to the generation and duration of EODs. Both of these microscopic techniques enable the imaging of optical sections through relatively thick specimens, such as vibratome sections with several tens of µm thickness through chemically fixed organs. To visualize the outline of the electrocytes for confocal fluorescence microscopy, specimens are labelled for plasma membrane proteins. For this purpose, we use Na^+^/K^+^-ATPase and plasma membrane Ca^2+^-ATPase (PMCA) as these two ion transporters are expressed in EOs at high level (Gallant et al. 2012; Lamanna et al. 2015; Cheng et al. 2024). An additional focus is the question of whether the various segments of the stalklet/stalk system differ by their equipment with these ion-transporting proteins, indicative of functional differences along the stalklet/stalk system.

## Materials and methods

### Samples

*C. compressirostris* were bred and raised at the University of Potsdam, Germany. Five male fish of 15.6 to 26.7 cm length were used for the study. All size measurements below are based on the EOs in adult fish of the length range of 20.7 to 21.7 cm (*N* = 3).

### Dissection and fixation of electric organs

The fish were euthanized by an overdose of clove oil (oleum caryophylli; Caesar & Loretz GmbH, Hilden, Germany), the caudal peduncle was cut off and submersed in physiological saline (114 mM NaCl, 2 mM KCl, 4 mM CaCl_2_, 2 mM MgCl_2_, 2 mM HEPES, 6 mM glucose, pH 7.2; Ban et al. 2015). The EOs were extracted, fixed with 3% paraformaldehyde in 0.1 M Na-phosphate buffer (pH 7.0) for 2 h at room temperature (RT), and washed extensively in 0.1 M Na-phosphate buffer. Alternatively, the entire peduncle, with the skin removed, was fixed, and EOs were dissected in Na-phosphate buffer after fixation. For vibratome sectioning, the EOs were cut into pieces of ~ 5 mm length by use of a micro-scalpel and embedded in 4% agarose (Biozym LE Agarose; Biozym Scientific GmbH, Hessisch Oldendorf, Germany) in phosphate-buffered saline (PBS). Specimens were sectioned in the transverse or the sagittal plane into about 200-µm-thick slices by use of a Pelco 101 Lancer 1000 vibratome sectioning system and Feather® double edge carbon steel blades (Ted Pella Inc., Redding, CA). Sections were collected in PBS and stained immediately afterwards (see below), or infiltrated with 50% glycerol in PBS and stored in this solution at − 20 °C until further use. For cryostat sectioning, ~ 5-mm-long pieces of EOs were infiltrated overnight with 25% sucrose in PBS, cryofixed in melting isopentane (approx. − 150 °C), sectioned at − 30 °C to a thickness of 25 to 30 µm in a Microm HM 550 MP (Thermo Fisher Scientific, Walldorf, Germany), collected on poly-l-lysine-coated coverslips, air-dried and stored at 4 °C until use.

### Immunofluorescence staining

Vibratome and cryostat sections were permeabilized with 0.01% Tween20 in PBS, treated with 50 mM NH_4_Cl, and washed with PBS. After treatment for 15–30 min with blocking solution (BS; 1% normal goat serum, 0.8% bovine serum albumin, 0.5% Triton X-100 in PBS), sections were incubated with primary antibody (see below) diluted in BS for 1–2 h at room temperature (cryosections) or 1 to 3 days at 4 °C (vibratome sections) in a humidity chamber, washed with PBS, reacted for 1 h at room temperature (cryosections) or overnight at 4° C (vibratome sections) with Alexa Fluor 568- or Cy3-conjugated secondary antibody, CF488A-phalloidin (Biotium Inc., Fremont, CA) and DAPI in PBS, and washed again extensively with PBS. Slices were mounted in Mowiol 4–88 embedding medium supplemented with 2% n-propyl gallate.

### Antibodies

Mouse monoclonal antibody α5 (10 µg/ml; DSHB at University of Iowa) against a highly conserved epitope of chicken Na^+^/K^+^-ATPase α-subunit (Takeyasu et al. 1988; Ban et al. 2015) was used to label Na^+^,K^+^-ATPase. This antibody has been validated to react with Na^+^/K^+^-ATPase α-subunit in teleost fish (e.g. Wilson et al. 2002; Mudsen et al. 2020) and is routinely used to localise Na^+^/K^+^-ATPase in various tissues, including EOs of *Eigenmannia virescens* (Ban et al. 2015) and of the mormyrid *Brienomyrus brachyistius* (Gallant et al. 2012). Plasma membrane Ca^2+^-ATPase (PMCA) was detected with mouse monoclonal antibody 5F10 (10 µg/ml; Sigma-Aldrich Chemie GmbH, Taufkirchen, Germany) against human erythrocyte PMCA (Borke et al. 1989). The epitope of this antibody, a 20 amino acid sequence (719–738 of human PMCA4b, FLCLEGKEFNRLIRNEKGEV; Caride et al. 1996), is highly conserved between isoforms and across a wide range of species, including the Pacific chub mackerel *Scomber japonicus* (Kwan et al. 2020) and the mormyrid *Brienomyrus brachyistius* (Gallant et al. 2012). BLAST sequence alignment demonstrates that this epitope shares 85% identity and 95% similarity with *C. compressirostris* PMCA isoforms expressed in the EO (Cheng et al. 2024). Synapses were identified with mouse monoclonal antibody SV2 (5 µg/ml; DSHB) against synaptic vesicle glycoprotein 2A from the EO of the elasmobranch *Discopyge ommata* (Buckley and Kelly 1985), and with rat monoclonal antibody mAb 35 (7 µg/ml; DSHB) against nicotinic acetylcholine receptor (nAChR) α1 subunit of *Electrophorus electricus* (Tzartos et al. 1981). Both SV2 and mAb 35 bind to epitopes that are highly conserved among vertebrate species (Kuscha et al. 2012; Tzartos et al. 1981). Secondary antibodies were obtained from ThermoFisher Scientific (Waltham, MA) and Jackson ImmunoResearch (Ely, UK).

### Imaging

An Axioskop (Carl Zeiss Microscopy Deutschland GmbH, Jena, Germany), equipped with a Plan-Neofluar × 10/0.3, a Plan-Neofluar × 20/0.5 and a Plan-Neofluar × 40/0.75 objective lens was used to image sections by differential interference contrast (DIC) microscopy and polarization microscopy. Fluorescence images were recorded with confocal microscopes LSM 710 and LSM 880 Airyscan (Carl Zeiss Microscopy GmbH, Jena, Germany), using a Plan-Apochromat × 20/0.8, a C-Apochromat × 40/1.20 W or a LD LCI Plan-Apochromat × 40/1.2 Imm objective lens. Alexa Fluor 568 (or Cy3) and CF488A were excited sequentially, whereas DAPI fluorescence was recorded in parallel with Alexa Fluor 568 (or Cy3). Alternatively, all dyes were excited in parallel and recorded by using online fingerprinting. Image processing was performed using ZEN 3.0, and figures were assembled in CorelDraw 2019. 3D reconstructions were created with the Fiji software.

## Results

### Organization of the electric organ

Figure 1 presents an overview on the organization of the EO in *C. compressirostris*. Each electrocyte is composed of a disc-shaped portion, also called the main body, and stalklets and stalks. The stalklets extend from the posterior side of the main body and fuse to stalks. These unite more and more to form a single main stalk that carries the site of innervation. In transverse sections through EOs of 20.7–21.7 cm–long animals, electrocytes had a roughly oval shape of about 5 mm × 2.5 mm, and covered thus an area of about 10 mm^2^. The site of innervation had a relatively central position on the electrocyte. Electrocytes were stacked at a distance (anterior face to anterior face) of about 175 µm. Their disc-shaped portion was about 15 µm thick and had an about 10–15 µm thick layer of collagen fibres close to its anterior face. Stalklets and stalks resided thus in an about 150 µm wide extracellular space between the posterior face of the electrocyte they belong to and the collagen layer anterior of the subsequent electrocyte.

### Differential distribution of Na^+^/K^+^-ATPase and PMCA

Transcriptome analysis in *C. compressirostris* has demonstrated that both Na^+^/K^+^-ATPase and PMCA have a far higher expression level in the EO than in skeletal muscle (Cheng et al. 2024). Since these ion transport ATPases are integral proteins of the plasma membrane, we suppose that labelling for these could possibly outline the surface of electrocytes, including their stalk and stalklet system. In order to test this hypothesis, we examined the subcellular distribution of PMCA and Na^+^/K^+^-ATPase α-subunit on sagittal cryostat sections through EOs.

**Fig. 2:**
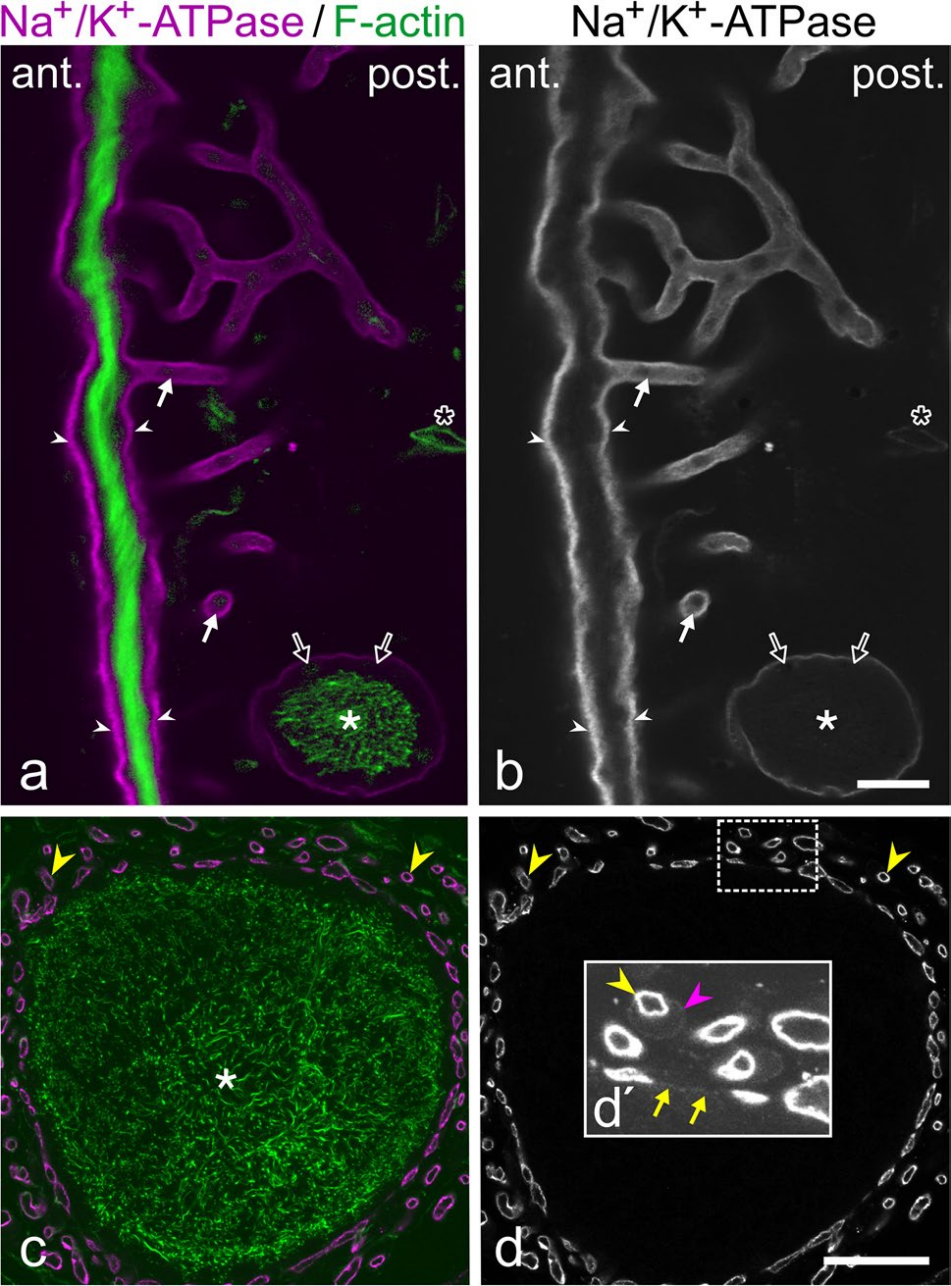
Distribution of Na^+^/K^+^-ATPase α-subunit on electrocytes. Sections were co-labelled with CF488A-phalloidin (green) to visualize the distribution of F-actin and to identify the main body of the electrocyte. **a**, **b** Sagittal section. Labelling for Na^+^/K^+^-ATPase (magenta in **a**, grey in **b**) is detected at high intensity on the anterior and posterior surface of the electrocyte main body (arrowheads), and on stalklets (arrows). Thicker stalks (asterisk) have faint labelling for Na^+^/K^+^-ATPase (open arrows). **c**, **d** Staining for Na^+^/K.^+^-ATPase (magenta in **c**, grey in **d**) on a cross-section through the innervated portion of a main stalk (asterisk). The area outlined by a dashed line in **d** is shown in the **inset d′** at higher magnification (× 2.5) and with the intensity increased to visualize faint fluorescence. Nerve fibres (yellow arrowheads) are intensely labelled. Staining is barely detectable on the main stalk membrane (yellow arrows in **d′**), similar to the staining of glial cells (magenta arrowhead in **d′**). Gamma correction was adjusted to 0.65 for the green channel (**a**, **c**) in order to visualize faintly labelled structures, such as blood vessels (open asterisk). Scale bars: 10 µm (**b**), 25 µm (**d**)

**Fig. 3:**
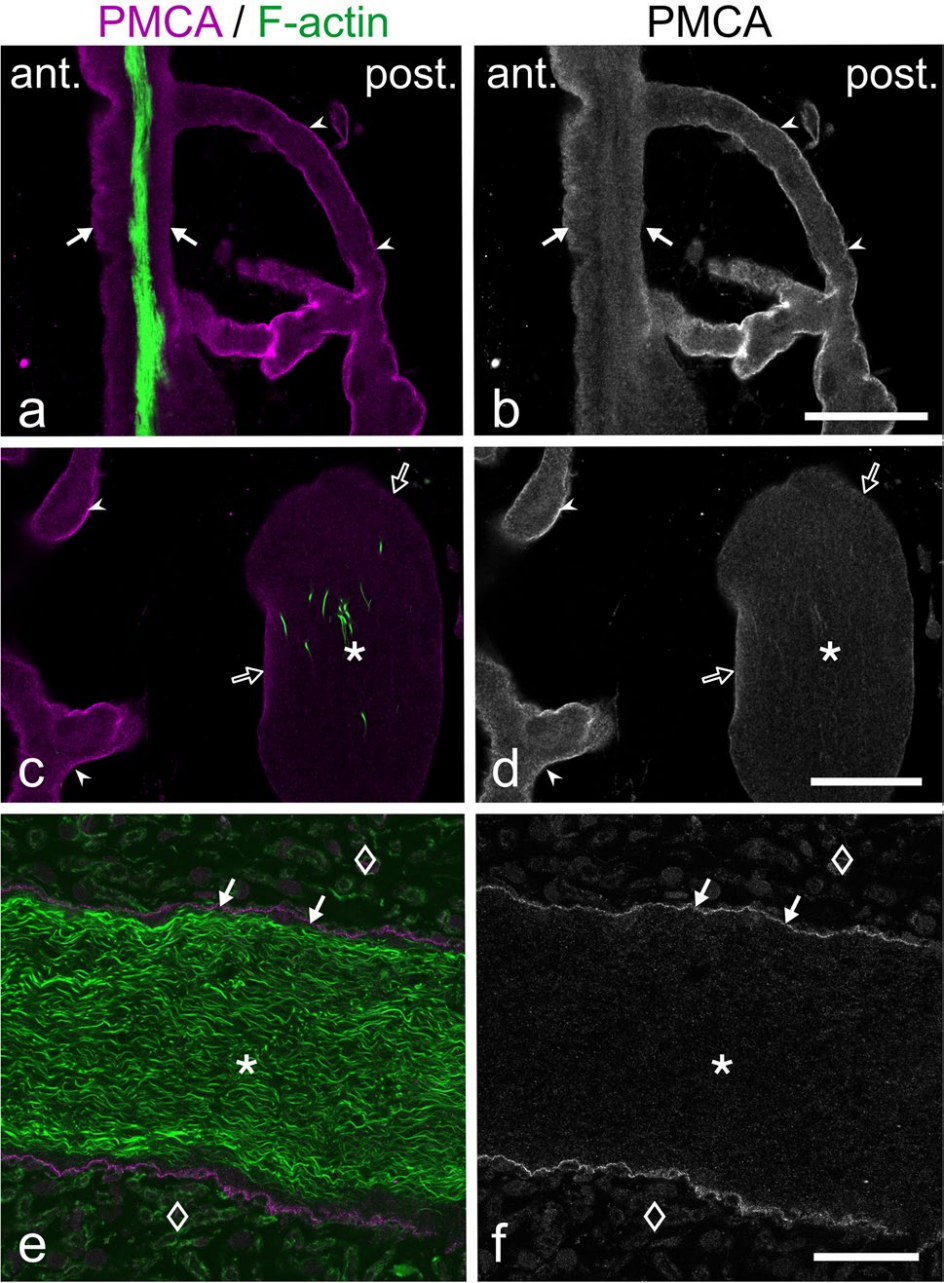
Distribution of plasma membrane Ca^2+^-ATPase (PMCA) on electrocytes. In order to identify the main body of the electrocyte and to distinguish the various portions of the stalklet/stalk system, sections were co-labelled with the F-actin probe CF488A-phalloidin (green). Labelling for PMCA is presented in magenta in **a**, **c**, **e**, and in grey values in **b**, **d**, **f**. **a–d** Sagittal sections. Anterior and posterior surface (arrows in **a**, **b**) of the main body and thicker stalks (asterisks in **c**, **d**) have weak labelling for PMCA (open arrows), whereas staining is enriched on stalklets (arrowheads). **e**, **f** Staining for PMCA on the innervated portion of a main stalk (asterisk) in longitudinal section. The plasma membrane is faintly labelled (arrows); the surrounding nerve fibres (diamonds) are without labelling for PMCA. Gamma correction was adjusted to 0.65 for the green channel in order to visualize faintly labelled structures. Scale bars: 10 µm (**b**, **d**), 25 µm (**f**)

**Fig. 4:**
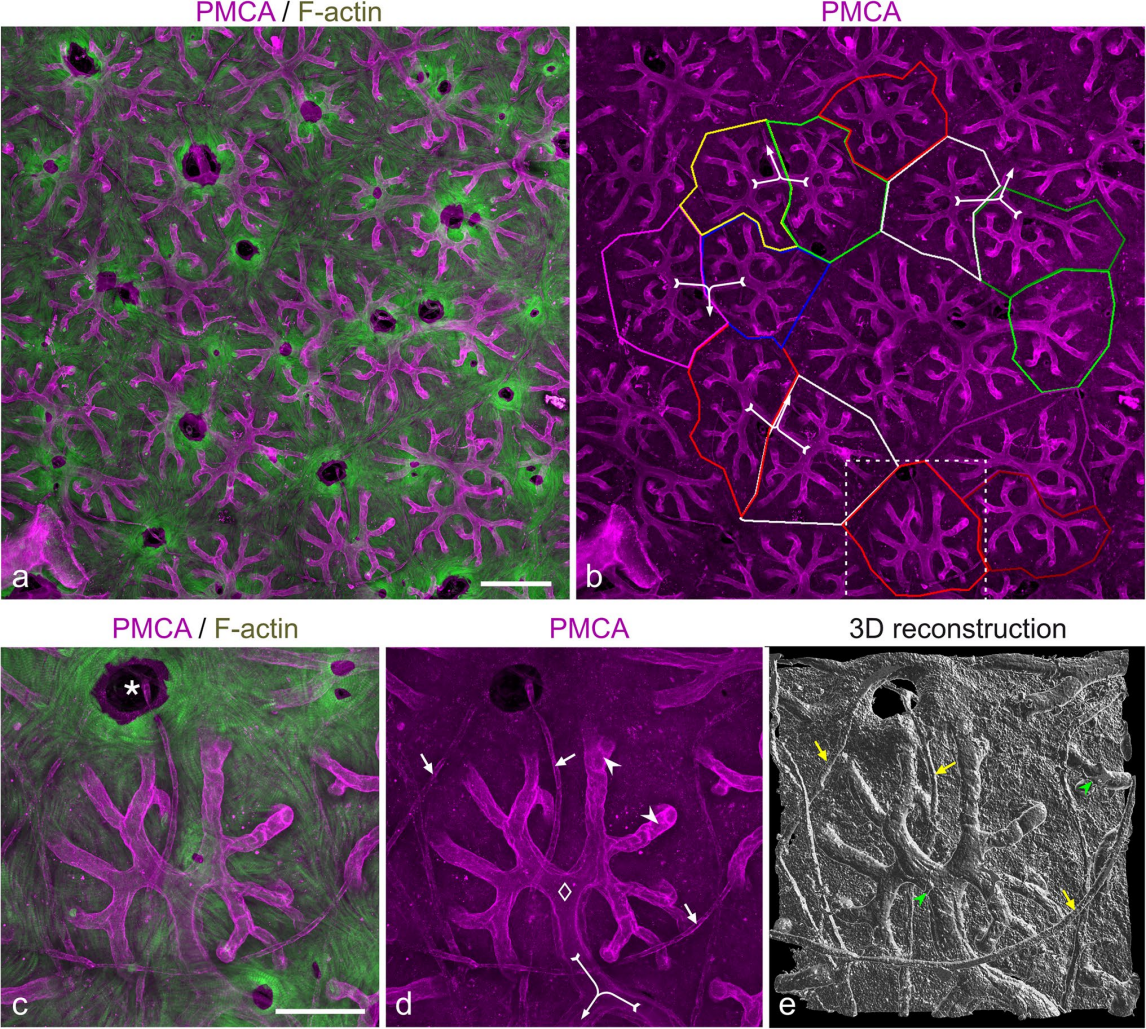
The stalklet system of electrocytes. Transverse vibratome sections (animal length 21.7 cm) were stained for plasma membrane Ca^2+^-ATPase (PMCA; magenta) and F-actin (green). Maximum intensity projections of ~ 41-µm-thick (**a**, **b**) and ~ 28-µm-thick (**c**–**e**) image stacks, with **e** presented as a 3D reconstruction. Note that PMCA is enriched in the stalklet membrane. Each 10 or more stalklets (arrowheads) extend from a polygonal area (outlined in different colours in **b**) and unite to a terminal stalk (diamond), with each two terminal stalks fusing to a preterminal stalk (uniting arrows). The stalklet system outlined at the bottom of **b** is shown at higher magnification in **c**–**e**. Arrows (white in **d**, yellow in **e**) indicate blood vessels stained for PMCA. Asterisk indicates a hole in the electrocyte permitting the passage of blood vessels. Green arrowheads in **e** indicate fibrocytes on stalklets. Gamma correction was set to 0.65 in the green channel. Note that the maximum intensity projection shown in **c** is presented as a movie with views from different angles (Movie S2). Scale bar: 100 µm (**a**, **b**), 50 µm (**c**–**e**)

In case of Na^+^/K^+^-ATPase α-subunit, intense staining was present on both the anterior and posterior face of the electrocyte main body (Fig. 2a, b). The more intense signal on the anterior side can be at least partially attributed to the organization of this membrane domain into short, narrow folds, resulting in a larger total membrane area. Furthermore, stalklets were labelled at an intensity similar to the posterior face of the main body. Labelling intensity gradually decreased upstream along the stalk system (Fig. 2a, b), being almost undetectable on the main stalk (Fig. 2c, d). In case of PMCA, the highest staining intensity was present on stalklets (Fig. 3a, b), whereas the stalk system including the main stalk and the anterior and posterior face of the electrocyte disc showed only faint labelling (Fig. 3a–f). In addition, blood vessels in the space between electrocytes were labelled for PMCA (Fig. 4d, e). We thus conclude that Na^+^/K^+^-ATPase and PMCA are differentially distributed on the stalklet/stalk system of electrocytes, with PMCA being concentrated on stalklets, but Na^+^/K^+^-ATPase being present along both stalklets and stalks, but almost exempting the main stalk.

### Structural organization of the stalklet system

**Fig. 5:**
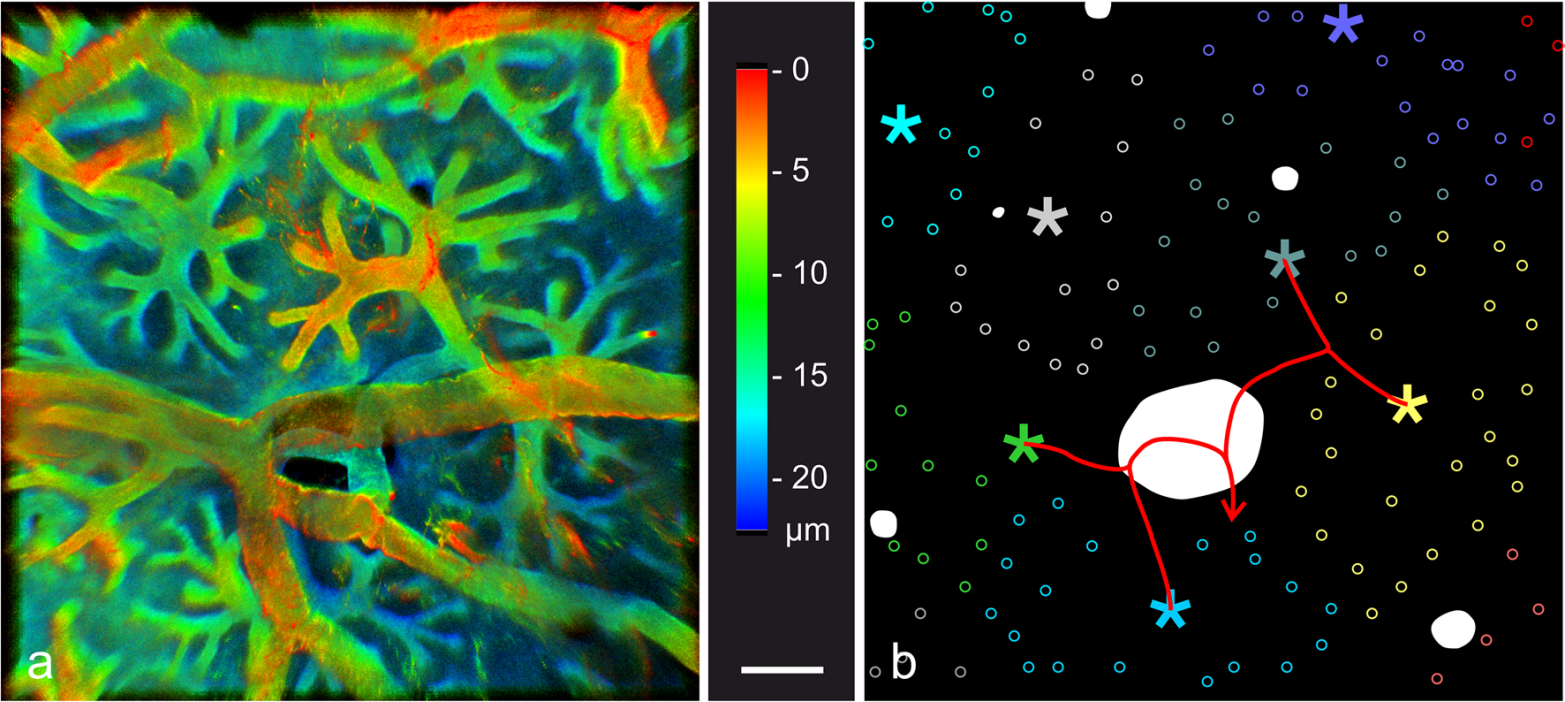
Organization of the stalklet system and the adjoining terminal and preterminal stalks. Transverse vibratome section (animal length 20.7 cm), stained for Na^+^/K^+^-ATPase α-subunit. **a** Depth-encoded maximum intensity projection of a 23-µm-thick image stack; **b** position of stalklets (circles) on the main body. Stalkets that form different asters are shown in different colours. Asterisks, position of the aster centre or stalk terminus, respectively. White areas, holes in the main body. Red lines, terminal and preterminal stalks connecting 4 stalklet asters. Note that the image stack is also available as a movie (Movie S1). Scale bar: 50 µm

The stalklet system was analysed by serial confocal imaging of transverse and sagittal vibratome sections stained with anti-PMCA and anti-Na^+^/K^+^-ATPase α-subunit, respectively. In maximum intensity projections of serial image stacks through anti-PMCA-stained specimens, the stalklet system stands out in high contrast against the electrocyte main body, permitting the analysis of the stalklet system within relative large areas (up to ~ 1 mm^2^) of a single electrocyte (Fig. 4). These images suggest that stalklets are arranged in a quite characteristic mode and a relatively regular pattern on the electrocyte posterior surface. Imaging of anti-Na^+^/K^+^-ATPase-stained sections visualized, besides the stalklets, the connecting stalk system and the surface of the main body (Figs. 5, S1; Movie S1), which made the 3-dimensional analysis more difficult and limited it to smaller areas or volumes, respectively.

**Fig. 6:**
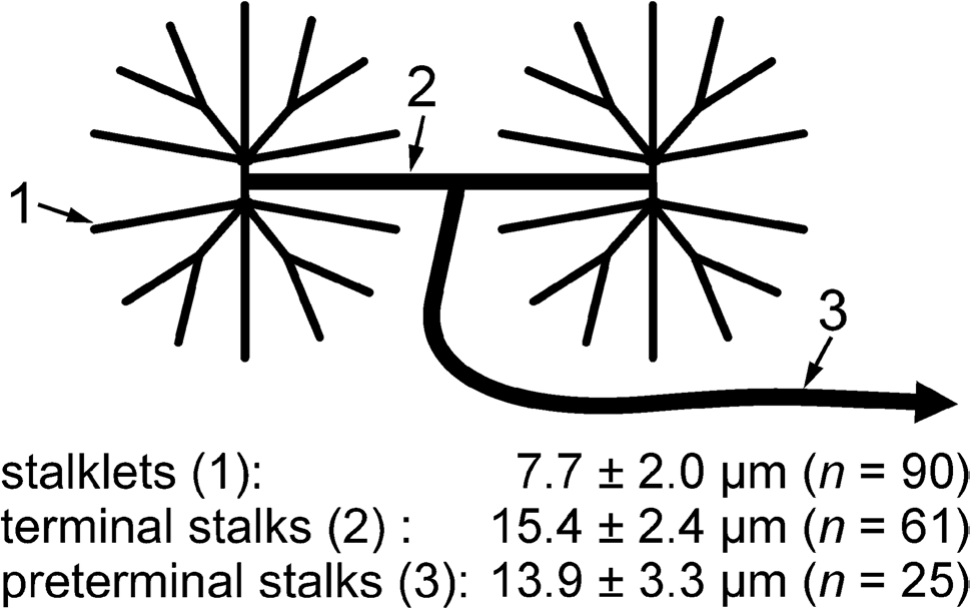
Stalklets and stalks are organized in a uniform mode. Each 10–20 stalklets (1) are arranged in an aster, each aster is attached to a short terminal stalk (2), and two terminal stalks then unite to a preterminal stalk (3). Diameters ($\overline{x }$ ± SD) of stalklets, terminal and preterminal stalks for fish in the size range 20.7 to 21.7 cm are presented. Note that diameters of terminal and preterminal stalks do not differ significantly (*P* = 0.081; Mann–Whitney rank sum test)

Stalklets measured 7.7 ± 2.0 µm ($\overline{x }$± SD; *n* = 90) in diameter and extended for about 50–100 µm between their attachment site on the electrocyte disc and the following stalk, termed terminal stalk (Figs. 4, 5 and 6). Each 10 up to 20 stalklets were arranged in an aster-like pattern, with the centre of the aster linked to the terminal stalk. Often stalklets merged halfway to the aster’s centre. Each aster covered a polygonal area of about 20 × 10^3^ µm^2^ and a diameter of 150–200 µm on the posterior face of an electrocyte (Fig. 4b). Only exceptionally, adjacent asters interdigitated with their stalklets. The stalklet asters rested relatively flat on the caudal surface of the electrocyte main body, with the aster centre positioned only 20–50 µm posterior of the later (Fig. S1; Movie S2). Attachment sites of stalklets were quite homogeneously distributed on the electrocyte, exempting only the area underneath the aster centre (Fig. 5b). Taking into account the density of stalklets on the main body (~ 7/10,000 µm^2^), the area covered by an aster (~ 20 × 10^3^ µm^2^), and the size of an electrocyte (~ 10 mm^2^), we calculate that each electrocyte has about 7000 stalklets organized in about 500 asters.

Terminal stalks had a diameter of 15.4 ± 2.4 µm (*n* = 61; Fig. 6) and were thus considerably thicker than stalklets. They extended for about 50 µm from the centre towards the periphery of the aster, and then fused with the terminal stalk of an adjoining stalklet aster (Figs. 4a, b, 5 and 6). The stalk formed by this fusion, termed preterminal stalk, had a diameter of 13.9 ± 3.3 µm (*n* = 25; Fig. 6) and therefore was in this respect not significantly different from the terminal stalk. The preterminal stalk then either fused after a short distance with another preterminal stalk, or extended for a larger distance towards and fused with a thicker stalk. Preterminal and terminal stalks were thus organized mostly in a dichotomic branching pattern.

### Organization of the stalk system

**Fig. 7:**
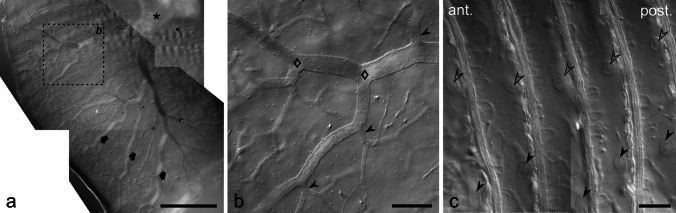
Layout of the stalk system of electrocytes. Vibratome sections in transverse (**a**, **b**) or sagittal orientation (**c**) through an EO were imaged by DIC microscopy. The main stalk (asterisk in **a**) branches in a dichotomic mode (diamonds in **b**) into thick stalks that extend towards the periphery of the electrocyte (arrows in **a**). On the way to the periphery, short thin stalks (arrowheads in **b**) branch off sideways. Thick stalks are arranged in a relatively uniform pattern on electrocytes, as cross-sections through thick stalks have similar positions on adjacent cells (arrowheads and open arrowheads in **c**). Scale bar: 500 µm (**a**), 100 µm (**b**, **c**)

Since the stalk system upstream of preterminal stalks could not be distinguished easily from other structures in low magnification views of anti-Na^+^/K^+^-ATPase-labelled vibratome sections (see Fig. 5a), we used DIC microscopy to analyse the organization of the stalk system from preterminal stalks up to the main stalk (Fig. 7).

The main stalk measured about 100 µm in diameter and was over a great extent covered by innervation (see below). On both sides next to the innervation site, the main stalk showed dichotomous branching, resulting in primary stalks with a diameter of ~ 80 µm. Stalks continued to branch in a dichotomous mode towards the periphery of the electrocyte, thus providing a branching system over the entire electrocyte, with stalk thickness gradually decreasing to about 20 µm. In sagittal vibratome sections, thick stalks occupied almost identical positions in the space between the electrocyte discs (Fig. 7c), indicating that the branching pattern was highly consistent for electrocytes in a column. Aside the dichotomous branching system, relatively thin (15–20 µm) stalks branched off to the side at irregular distances (Fig. 7b) and ramified into preterminal and terminal stalks. The main stalk and the subsequent daughter stalks were without thin side branches. The central area of the electrocyte was thus connected to the innervation site by stalks that branched off the stalk system at some distance towards the periphery of the electrocyte, and then took a detour in direction of the main stalk.

DIC microscopy visualized further some structural details of the stalks (Fig. S2). These resided in a voluminous extracellular space and had few star-shaped cells on their outer surface, likely fibrocytes. Nuclei were situated just below the stalk surface, and the core of the stalk contained fibrous material along with few organelles. Since these organelles had a relatively high refractive index, they likely represent mitochondria or vesicular structures with protein or lipid content. Polarization microscopy demonstrated further that the main stalk and thick stalks exhibit positive birefringence (Fig. S3), indicating an ordered organization of the fibrous core.

### Innervation site of the electrocytes

The innervation site on the main stalk was analysed by DIC microscopy, and by immunofluorescence labelling for Na^+^/K^+^-ATPase α-subunit, synaptic vesicle glycoprotein 2A (SV2) and nicotinic acetylcholine receptor (nAChR) α1 subunit. It is to be assumed that nerve fibres can be identified by intense labelling for Na^+^/K^+^-ATPase (Fig. 2d), whereas SV2 and nAChR represent marker proteins for synapses of electromotoneurons and electrocytes.

**Fig. 8:**
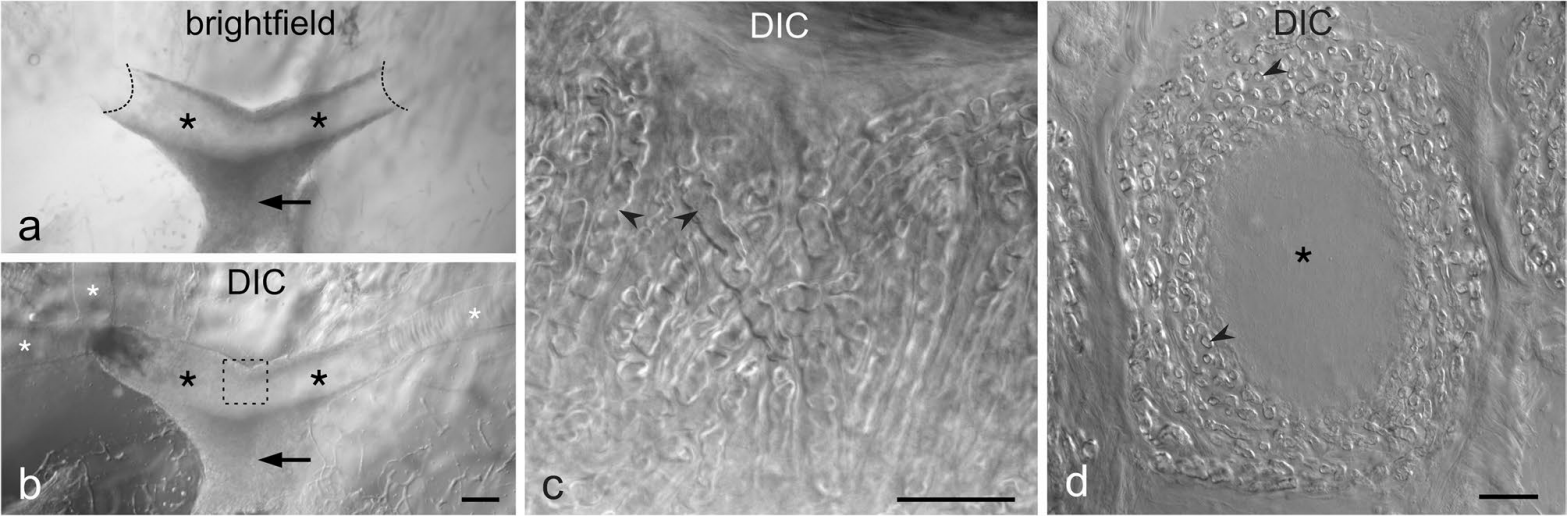
Innervation site. **a**, **b** Transverse vibratome section through an EO. The nerve (arrow) extends towards and reaches around the main stalk (black asterisks). The brightfield image (**a**) visualizes the boundaries (dashed lines) of the innervated main stalk segment; the DIC image (**b**) visualizes adjacent non-innervated stalk portions (white asterisks). **c** DIC image of an area as indicated by the dashed square in **b**. Numerous nerve fibres (arrowheads) surround the main stalk at the site of innervation. **d** Cross-section (~ 25-µm-thick cryosection) through a main stalk at the site of innervation. The main stalk (asterisk) is completely surrounded by a thick layer of nerve fibres (arrowheads). Scale bars: 100 µm (**a**, **b**), 25 µm (**c**, **d**)

**Fig. 9:**
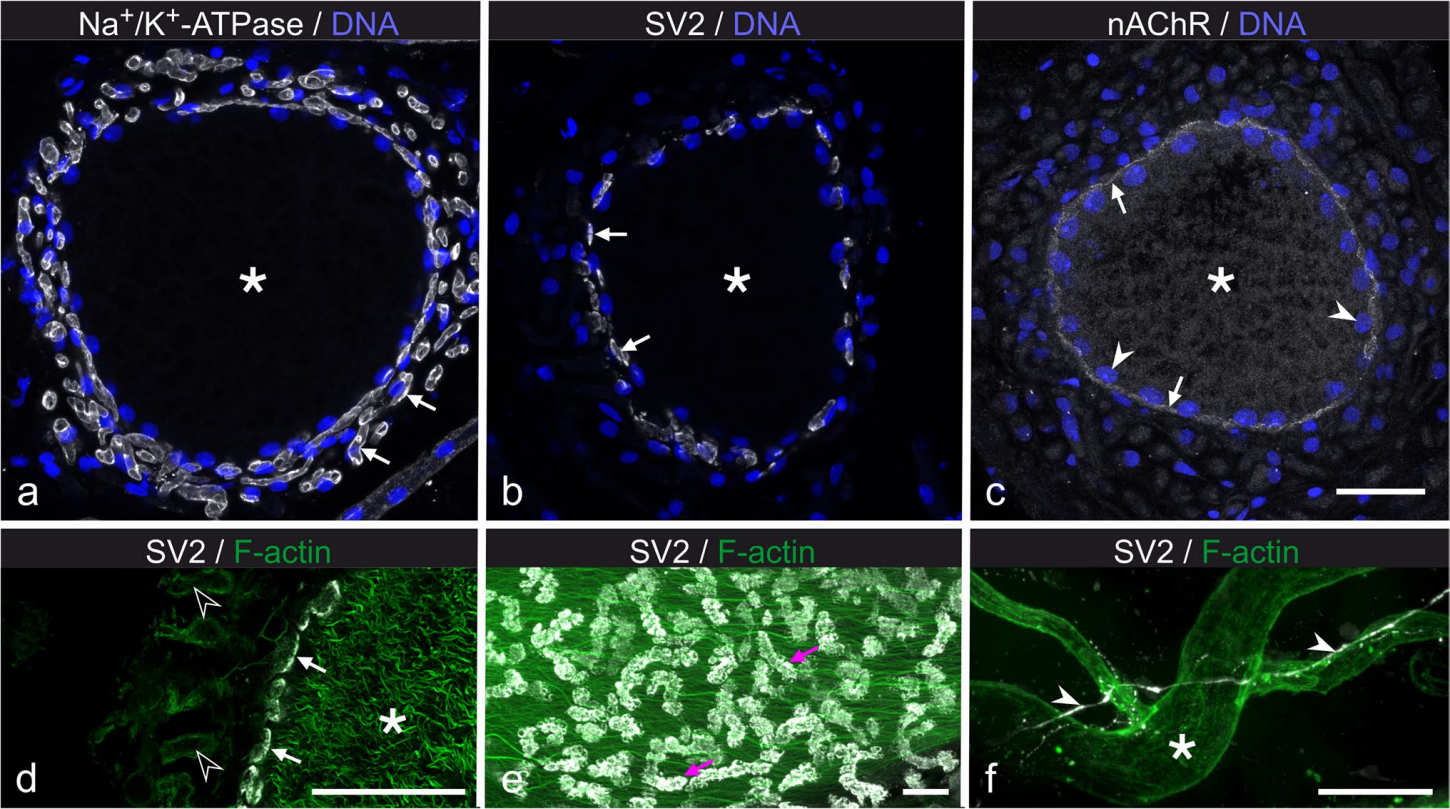
Synapses on the main stalk. **a**–**c** Cryosections stained for Na^+^,K^+^-ATPase α-subunit (grey values in **a**), synaptic vesicle glycoprotein 2A (SV2; grey values in **b**) and nicotinic acetylcholine receptor (nAChR; grey values in **c**). All sections were co-stained with DAPI (blue). Images are presented as maximum intensity projections of 3.15 to 3.35 µm thick stacks of optical sections. Staining for Na^+^,K^+^-ATPase outlines nerve fibres (arrows in **a**) around the main stalk (asterisk), whereas staining for SV2 visualizes presynapses (arrows in **b**). The surface of the main stalk exhibits staining for nAChR (arrows in **c**). Note the subsurface position of nuclei in the main stalk (arrowheads in **c**). **d** Single optical plane through the innervated region of a main stalk (asterisk) at higher magnification. SV2-positive presynapses (arrows) are associated with the surface of the main stalk. Faint labelling for F-actin (green; open arrowheads) outside of the main stalk indicates nerve fibres and likely the surrounding Schwann cells. **e** Maximum intensity projection of a 10.3 µm thick image stack at the site of innervation, stained for SV2 (white) and F-actin (green). The main stalk extends in horizontal direction and is highlighted by F-actin bundles. The stalk surface is covered by numerous SV2-positive presynapses (magenta arrows). **f** SV2-positive labelling (white) is detected in neuronal fibres (arrowheads) associated with blood vessels (asterisk) that reside in the extracellular space between electrocytes. Gamma correction was set to 0.66 (**e**, **f**) or 0.50 (**d**) for the green channel. Scale bars: 25 µm (**a**–**c**), 10 µm (**d**–**f**)

A 150–200-µm-thick branch of the spinal nerve approached the main stalk of each electrocyte (Fig. 8a, b). The nerve ending embraced the main stalk completely over a length of ~ 1 mm. At this site, numerous nerve fibres with a diameter of 1–3 µm and ensheathed by Schwann cells, the later identified by weak labelling for F-actin, formed a 20–30 µm thick layer around the main stalk (Figs. 8c, d and 9a, d). Only fibres next to the stalk surface were positive for SV2 (Fig. 9b, d, e), indicating that these sites represent presynapses. The SV2-positive sites covered roughly 50% of the main stalk surface (Fig. 9e). Correspondingly, a large fraction of the surface of the main stalk was labelled for nAChR (Fig. 9c).

Besides on the main stalk, few SV2-positive fibres were detected in the space between electrocytes. These fibres were in close contact to blood vessels, running alongside for extended stretches (Fig. 9f). It may thus be presumed that yet to be identified neurotransmitters released by these fibres are involved with the regulation of blood flow within the EO.

### F-actin in the stalk system

**Fig. 10:**
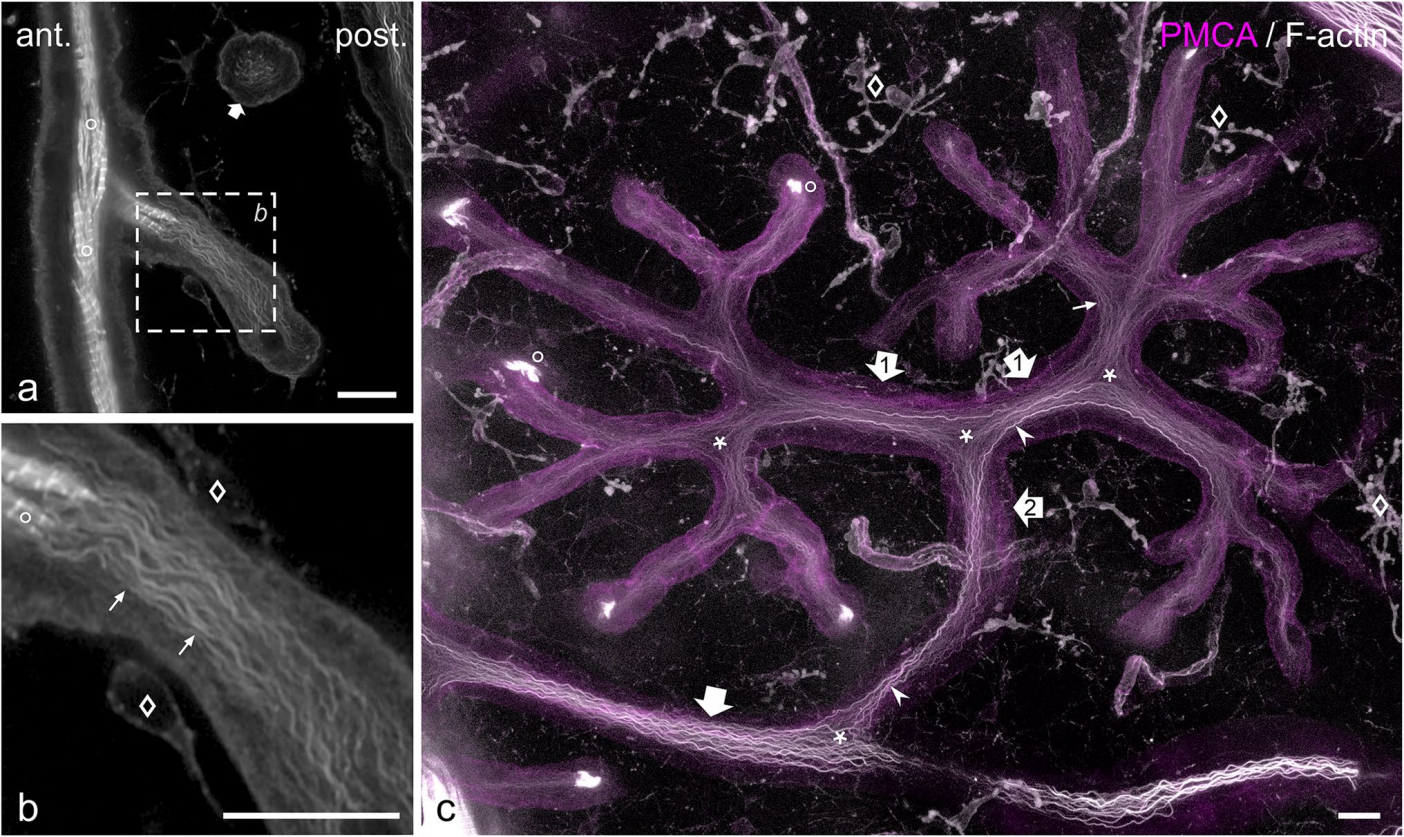
F-actin in stalklets. Sagittal (**a**, **b**) and transverse (**c**) vibratome sections through EOs were stained with AF488-phalloidin **(a**–**c**, white) and anti-PMCA (**c**, magenta). Gamma correction was set to 0.45 for the white channel. **a**, **b** Single optical plane; **c** maximum intensity projection of a ~ 10.0 µm thick image stack. The myofibrillar system (circles) in the electrocyte extends into the base of the stalklet and then splays into (likely) single filaments (arrows in **b**). The filaments extend over the entire length of the stalklets and continue to extend into the terminal stalks (broad arrows #1 in **c**) and the preterminal stalk (broad arrow #2 in **c**). In terminal and preterminal stalks, actin filaments form thin bundles, as indicated by more intense labelling (arrowheads), and these extend further into the upstream stalk system (broad arrow in **c**). Note that actin filaments also form triangular arrays (asterisks) at fusion sites of two stalklets or two terminal stalks. Broad arrow in **a**, cross-sectioned stalklet; diamonds in **b**, **c**, fibrocytes on the stalklet surface and between stalklets. Scale bars: 10 µm

In order to highlight the outline of cells for fluorescence microscopy, we routinely co-label tissues with fluorescent phalloidin, since F-actin is often enriched at the plasma membrane (e.g. Zimmermann et al. 2003; Klose et al. 2017). In the EO, F-actin was detected in the electrocyte main body in a regular, sarcomere-like pattern (Figs. 4a, c and 10a). This finding is in line with the identification of myofibrills in mormyrid EOs by transmission electron microscopy (Bruns 1971; Schwartz et al. 1975; Bass et al. 1986; Korniienko et al. 2021). Contrary to our expectation, however, F-actin was not enriched at the plasma membrane of stalklets and stalks. Instead, we detected a prominent F-actin system in the core of these structures.

At the base of stalklets, filamentous structures branched off the myofibrillar actin system and run along the inside of the stalklets (Fig. 10a, b). In some cases, the myofibrillar assemblies extended for a few micrometres into the base of stalklets before splaying into numerous filamentous structures. Based on the weak and quite uniform intensity of phalloidin labelling, we suppose that these filamentous structures within stalklets represent single actin filaments. These extended over the entire length of the stalklets into the terminal stalks (Fig. 10c). When two stalklets fused on their way to the terminal stalk, actin filaments extended into the common stalklet, but others also into the sister stalklet, thus forming triangular F-actin assemblies at stalklet branchpoints (Fig. 10c). Within terminal and preterminal stalks, some F-actin structures were labelled at higher intensity, suggesting that actin filaments joined to form bundles (Fig. 10c).

**Fig. 11:**
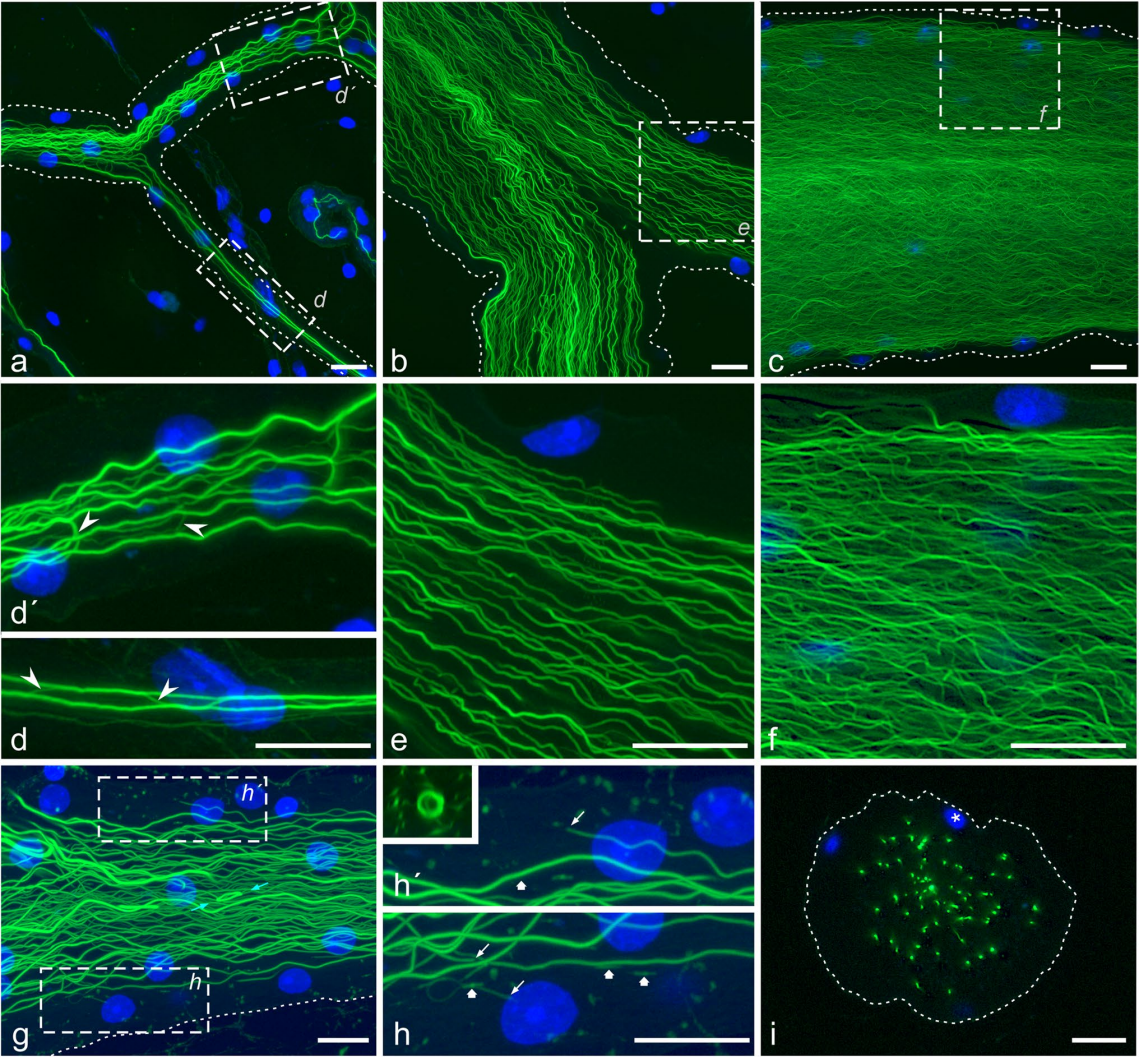
F-actin in stalks. Vibratome sections were stained with AF488-phalloidin (green) and DAPI (blue). **a**, **d** Thin stalks; **b**, **e**, **g**–**i** thick stalks; **c**, **f** main stalk. **a**–**h** Maximum intensity projections of ~ 9.4 µm (**a**, **d**), ~ 5.9 µm (**b**, **e**), ~ 4.0 µm (**c**, **f**) and 33.4 µm (**g**, **h**) thick image stacks; **i** single optical section. Dashed lines in **a**–**c**, **g, i** outline the contour of the stalk, deduced from slight difference in fluorescence intensity between the cytoplasm and the background (extracellular space). Boxes in **a**–**c**, **g** indicate areas shown at higher magnification in **d**–**f**, **h**. In all images, gamma correction was set to 0.45 for the green channel to visualize faintly labelled F-actin structures. **a**–**f** Thin stalk, thick stalks and main stalks have increasing numbers of F-actin bundles. F-actin bundles rarely split / fuse or turn backwards (arrowheads in **d**, **d′**). Moreover, F-actin bundles may terminate (arrows in **h, h′**) and fragments of F-actin bundles (broad arrows in **h**, **h′**) are detected especially in the subsurface region. Cyan arrows indicate a circular F-actin structure that is also presented in the inset (**h′**) as a calculated xz section. **i** Cross-section through a stalk. F-actin bundles reside in the centre, nuclei (asterisk) at the periphery. Note that the image stack of **g** is also presented in form of yz sections in Movie S3. Scale bars: 10 µm

As the stalk diameter increased in upstream direction towards the main stalk, the number and the density of these F-actin bundles increased in parallel (Fig. 11). Within the stalks, F-actin bundles were confined to the core, exempting the nuclei-containing subsurface region (Fig. 11i). Most of these F-actin bundles had a virtual diameter of about 0.2 µm, which is the resolution limit of our microscopic setup. Since the intensity of the F-actin assemblies with identical virtual diameter varied to a great extent (Fig. S4), we conclude that these structures represent compact bundles of various numbers of actin filaments. The F-actin bundles did not run completely straight along the stalk’s long axis, but were wavy, did branch, ended blindly or made even 180° turns (Fig. 11d, g, h). Position and distance of the F-actin bundles to each other was highly variable within a stalk segment (Movie S3). In addition, F-actin structures with a length of only a few micrometres were detected at the stalk core periphery in the subsurface layer (Fig. 11h). We suppose that these F-actin bundles in the core of stalks correspond to the filamentous structures observed by DIC microscopy.

## Discussion

### Methodical aspects

Primary goal of our study was the analysis of the 3-dimensional layout of the stalklet/stalk system of an electrocyte by light-microscopic techniques. This task should be achieved by (1) physical slicing of EOs to obtain sections that contain an entire electrocyte including its stalklet/stalk system and (2) selective staining of stalklets and stalks to distinguish them from other cellular structures within the EO e.g. blood vessels and fibrocytes. On the way to achieve our goal, we had to face several problems and to make some compromises. First, no probe entirely specific for stalklets and stalks was available. Although anti-PMCA labelling was highly enriched on stalklets, there was only faint labelling on stalks whereas blood vessels were fairly well labelled. Anti-PMCA labelling was thus quite well suited for the analysis of the stalklet system, but not for the stalks. Anti-Na^+^/K^+^-ATPase, on the other hand, stained stalklets and stalks, but the anterior and posterior membrane of the electrocyte disc at even higher intensity, and the main stalk only insignificantly. Due to the extent and complexity of the structures labelled with anti-Na^+^/K^+^-ATPase, only small volumes of the EO could be analysed by use of this antibody. Second, the EO is relatively inhomogeneous in its optical properties. The electrocyte main body and the layer of collagen fibres exhibit a high refractive index, but the space in which stalks and stalklets reside has a relatively low refractive index. It thus turned out that an ideal transverse section for imaging the stalklet/system should include a single electrocyte, with the cutting plane between the stalk system and the collagen layer posterior of it, and section should be mounted with the posterior side facing the coverslip and objective lens. In this orientation, the stalklet/stalk system can be imaged without the need to focus through the collagen layer and/or electrocyte disc as these structures destroy the optical homogeneity and absorb a considerable amount of light. Since electrocytes are extremely large in size, and the disc portion is not completely flat over its entire extent, it is practically impossible to obtain sections of exactly one-cell-thickness, including an entire electrocyte and with the section plane exactly between stalks and collagen layer. We were thus limited in this study by the fact that larger cell areas fulfilled these ideal conditions only by chance. It was possible to visualize the stalklet system within a maximal area of about 1 mm^2^, corresponding to ~ 10% of the size of an electrocyte. Moreover, whereas stalklets and terminal stalks could be visualized by anti-PMCA and anti-Na^+^/K^+^-ATPase labelling in conjunction with confocal optical sectioning, the layout of the upstream stalk system and of the main stalk could be analysed by DIC microscopy only.

It may be supposed that isolation and staining of individual electroytes may be advantageous for analysing the 3D-structure of the stalklet/stalk system of an entire electrocyte. Indeed, confocal microscopy has been used previously to analyse the morphology of isolated *Paramormyrops kingsleyae* electrocytes, at least at low magnification (Gallant et al. 2011). In that study, however, cells were far smaller (~ 1 mm × 0.5 mm) than electrocytes in adult *C. compressirostris*. We suppose that dissection of single electrocytes of adult *C. compressirostris* EOs may destroy or at least distort the delicate 3-dimensional stalklet/stalk system, and that our technique provides a view that corresponds much more closely to the morphology in situ.

### PMCA and Na,K-ATPase are differentially distributed on stalklets and stalks

EOs are specialized for the generation of action potentials. They therefore require a relative high ion transport activity to maintain ion gradients and excitability. This need is paralleled by the expression of Na^+^/K^+^-ATPase α- and β-subunits at a level far higher than in skeletal muscle (Gallant et al. 2012; Lamanna et al. 2015; Cheng et al. 2024). Moreover, PMCA is expressed at a higher level in EO than in skeletal muscle (Gallant et al. 2012; Cheng et al. 2024). We have used antibodies against well conserved epitopes and not selective for particular paralogs or splice variants to probe the distribution of both ion pumps in the EO.

The finding that Na^+^/K^+^-ATPase is quite prominent on the anterior and posterior surface of the electrocyte is not surprising. Action potentials generated at both membrane domains are associated with sodium inward and potassium outward currents, and both membrane domains therefore require high Na^+^/K^+^-ATPase activity to re-establish and maintain Na^+^ and K^+^ transmembrane gradients. The more intense labelling of the anterior face is at least partially due to the organization of this membrane into short narrow folds that can be resolved with high resolution imaging techniques only (data not shown). In stalklets and stalks, the amount of Na^+^/K^+^-ATPase appears to gradually decrease in upstream direction, being almost undetectable on the main stalk. Similarly, in the EO of the mormyrid *Brienomyrus brachyistius*, in which also both faces produce an action potential (Carlson 2002), Na^+^/K^+^-ATPase is present on both sides of the electrocyte to a similar amount, whereas no staining was visible on stalks (Gallant et al. 2012). The relatively low amount of Na^+^/K^+^-ATPase on stalks may thus be a common feature of mormyrid electrocytes. This finding may indicate a low need for this ion pump on the stalk system, possibly due to relatively low transmembrane Na^+^ and K^+^ fluxes at these membrane areas during electrocyte excitation (see below). Alternatively, Na^+^/K^+^-ATPase molecules on the stalk system may have a higher activity compared to Na^+^/K^+^-ATPase on the electrocyte disc, either by expression of different isoforms or by regulatory mechanisms, such as associated proteins or phosphorylation (Therien and Blostein 2000).

Stalklets differ from the stalk system not only by the more intense staining for Na^+^/K^+^-ATPase but also for PMCA. In the latter case, the differential distribution is even more pronounced, with PMCA being enriched on the stalklets vs. the stalk system and the anterior and posterior face of the electrocyte disc. Based on this finding we suppose that Ca^2+^ and its transients fulfil important but yet unknown functions in stalklets. Remarkably, transcriptome analysis of *C. compressirostris* EO demonstrated overexpression of several genes involved in Ca^2+^ signalling, e.g. Ca^2+^-binding proteins parvalbumin 9 and S100b, Ca^2+^/calmodulin-dependent protein kinase and inositol 1,4,5-trisphosphate receptor Ca^2+^ channel (Cheng et al. 2024). It may be interesting to determine whether any of these proteins is also enriched in stalklets. Taken together, our findings strongly indicate that the stalklet/stalk system is not uniform in its physiological properties.

### Morphology of stalk/stalklet system and its possible impact on the EOD

It has been known for long that the morphology of electrocytes is a critical factor in the shaping of the EOD. However, the focus as yet was on the geometry of the stalk system, in particular, on whether and how often stalks penetrate the electrocyte disc (Bass 1986a; Alves-Gomes and Hopkins 1997; Gallant et al. 2011). These characteristics determine the number and polarity of the phases of the EOD. The 3D layout of the stalk system and its possible impact on the EOD have not been analysed yet in detail in any mormyrid species. It is supposed that the morphology of stalks and stalklets is a critical component in the shaping of the EOD as the excitation initiated at the innervation site needs to be propagated along and spread over the stalk system to reach and initiate spikes at the disc in a synchronized mode over the entire area (Bennett 1971). Coordinated depolarization of the entire disc area and thus the organization of the stalk system may be especially critical in species with a relatively short EOD. *C. compressirostris* is ideally suited to examine how the stalklet/stalk system is adapted to achieve a synchronized depolarization of the electrocyte disc since the EOD with less than 0.2 ms is one of the shortest among mormyrid species (Hopkins 1981; Paul et al. 2015; Kirschbaum et al. 2016).

Above results demonstrate that the stalklet/stalk system in adult *C. compressirostris* is organized in a characteristic, relatively fixed pattern. The main stalk is of large calibre (~ 100 µm) and associated with hundreds of synapses covering an extensive area on its surface. The innervation site on the main stalk is positioned quite centrally on the electrocyte, thus ensuring similar lengths of stalks on the dorsal and ventral half of the electrocyte. The subsequent stalk system branches in a mostly dichotomic mode, with the diameter gradually decreasing towards the periphery of the electrocyte, and stalk position being largely conserved between electrocytes. The central area of the electrocyte is connected to the innervation site by stalks making a detour and thus by a stalk system of similar total length than to the electrocyte periphery. This can be also observed on the isolated stalk system of *C. compressirostris* (Fig. 61c in Bennett 1971). Preterminal stalks with a diameter of about 14 µm branch off along the stalk system, and split up into two terminal stalks of similar thickness. The latter are connected to an array of stalklets of similar length that radiate around the stalk terminus to join the posterior face of the electrocyte disc.

As for unmyelinated axons (Pumphrey and Young 1938), the propagation velocity for action potentials in the stalk system should increase with diameter with close to a square root dependence. Since action potentials have been recorded on the electrocyte stalk in mormyrid EOs (Bennett and Grundfest 1961; Bennett 1961, 1971), it may be supposed that postsynaptic potentials elicited at the site of innervation initiate action potentials at or close to the main stalk. The large calibre of stalks should ensure that these action potentials are propagated at high velocity from their site of origin radially over the entire electrocyte, thus keeping differences in conduction time between different stalks low. Dichotomic arborization, ensuring similar propagation velocities in the daughter stalks, and similar length of the stalk system to the electrocyte centre and periphery may support synchronization of action potentials. The stalk system in *C. compressirostris* is thus ideally organized to ensure that action potentials are propagated from the site of innervation over the entire posterior face with minimal latency variation among different stalks. The downstream component with lowest diameter and accordingly lowest propagation velocity, the stalklets, are quite similar in diameter and length and evenly distributed over the electrocyte disc, decreasing the probability of a temporal spread between action potentials along different stalklets. Since the stalklets radiate around the terminal stalk with an orientation mostly parallel to the disc, propagation of depolarization along the stalklets is involved with a current flow at right angle to the posterior-anterior axis, should thus not contribute to the EOD in a significant mode, and ensure a sudden onset of the EOD.

 Puzzling in our results, however, is the low staining intensity for Na^+^/K^+^-ATPase on thick stalks and the main stalk. Since action potentials are associated with enormous Na^+^ and K^+^ fluxes across the membrane, it may be expected that Na^+^/K^+^-ATPase is present at high density, as it is the case for the posterior and anterior face of the disc portion, and the electromotoneuron nerve fibres around the main stalk (Figs. 2d and 9a). It may thus be considered that an excitatory postsynaptic potential (EPSP) generated on the main stalk spreads passively over a greater distance along the stalk system to a downstream site of spike initiation. Although spike depolarizations have been recorded on stalks of mormyrid electrocytes, it is neither clear whether this applies to the EO in all mormyrid species, or at which site the electrode was placed exactly along the stalk system. The mode of innervation with numerous synapses covering an enormous area on the main stalk may support the possibility of spike initialization away from the innervation site. Simultaneous activity of all synapses should produce an EPSP of large amplitude that can spread over a large distance. Moreover, a high membrane resistance, the inverse of the permeability, is another parameter that influences the propagation of excitation. A high membrane resistance results in a large length constant and thus the spread of depolarization over a great distance. The relatively low staining intensity for Na^+^/K^+^-ATPase on thick stalks may suggest a low need for active transport of K^+^ to compensate for K^+^ leak currents, and thus a low channel density and high membrane resistance, respectively. The thick diameter of the main and downstream stalks and the dichotomic branching pattern may equally support the spread of an EPSP. These features together, postsynaptic potential of large amplitude, high resistance of the stalk membrane, large diameter of the stalks, and uniform branching pattern, could ensure that EPSPs can spread over a considerable extent of the stalk system and reach the threshold level at all downstream initiation sites at (almost) identical times.

In order to distinguish between above scenarios, information on the exact location of spike initiation site(s) is required e.g. by electrophysiological techniques or by immunolocalization of voltage-gated Na^+^ channels.

### Internal organization of the stalk/stalklet system

Both the stalks and the main body of the electrocyte contain prominent cytoskeletal structures of F-actin, but of different organization. In the main body, the F-actin system shows a regular cross-striated pattern, in accordance with electron microscopic images of electrocytes (e.g. Bruns 1971; Schwartz et al. 1975; Bass et al. 1986; Korniienko et al. 2021) and similar to the myofibrillar system in skeletal muscle cells. In stalks, however, the F-actin system is without any cross-striations but consists of numerous long F-actin bundles, extending longitudinally in the core of the stalks. To our knowledge, there have been as yet no observations by electron microscopy of such cytoskeletal elements in mormyrid electrocytes. Since the phalloidin labelling intensity differs to a large extent among these actin structures, we conclude that they are composed of variable numbers of closely packed actin filaments. Although we do not know whether actin filaments within an individual bundle are arranged in a unipolar or bipolar mode, the finding that some bundles make 180° turns suggests that at least actin filaments in adjacent bundles can be of different polarity.

The structural differences in the actin cytoskeleton between electrocytes and skeletal muscle cells, and the different F-actin systems within an electrocyte are paralleled by the expression of different actin-binding proteins. Expression of some sarcomere-specific proteins, such as skeletal muscle myosin-2, troponin, tropomyosin, titin and nebulin, is downregulated in the EO of *C. compressirostris* (Cheng et al. 2024), being in line with ultrastructural differences between myofibrils in both cell types (Korniienko et al. 2021). In contrast, genes encoding for molecular components of the Arp2/3 complex, the F-actin-uncapping protein CARMIL1, gelsolin, the actin remodeling regulator NHS, non-muscle myosin-2 and several unconventional myosins (1d, 1e, 3b and 15) are upregulated in the EO (Cheng et al. 2024). The Arp2/3 complex, CARMIL1 and gelsolin are implicated in the control of F-actin polymerization (Pollard 2016). Unconventional myosins are involved in the transport of membranous organelles along actin filaments, but may also play a role in F-actin organization and dynamics (Woolner and Bement 2009; Fang et al. 2015). We suppose that at least some of these actin-binding proteins with upregulated expression level are involved in the assembly and/or organization of the actin system which is unique for electrocytes, namely the F-actin bundles within stalks. The presence of short F-actin structures at the periphery of the bundle arrays, likely fragments of F-actin bundles or nucleation sites for new bundles, is in line with a dynamic behaviour of the F-actin system in stalks, and with the overexpression of proteins involved in F-actin assembly/disassembly.

What is the function of these peculiar F-actin bundles in stalks? We suppose that due to the large size and complex shape, and the location within a space with apparently little external support by other cells and extracellular fibres, stalks may require additional stabilization by cytoskeletal elements, as suggested already by Gallant et al. (2012). In addition, the F-actin bundles may provide a railway for the transport of material along the extensive stalk system.

## Concluding remarks

By immunofluorescence staining of the plasma membrane proteins Na^+^/K^+^-ATPase and PMCA in conjunction with confocal microscopy, this study provides novel details on the structural organization of electrocytes in *C. compressirostris*. The methods presented here can now be applied to EOs of different mormyrids and of various ages in order to analyse the contribution of stalk/stalklet organization and of the subcellular distribution of ion transporters and ion channels on EOD signal diversity. We also suppose that these techniques can be easily adapted for use on EOs of Neotropical knifefishes (Gymnotiformes), another group of electric fish with an enormous variety in EOD waveform and electrocyte morphology (Bennett 1971; Bass 1986a, b; Crampton et al. 2013; Markham 2013).

## Supplementary Information

Below is the link to the electronic supplementary material.

Supplementary file1 (AVI 4178 KB)

Supplementary file2 (AVI 13716 KB)

Supplementary file3 (AVI 15054 KB)

Supplementary file4 (PDF 596 KB)

## Data Availability

All relevant data are included in the manuscript. Raw data are available from the corresponding author upon reasonable request.
