# Supplementary material for: Organization of the stalk system on electrocytes in mormyrid weakly electric fish Campylomormyrus compressirostris
Source: Cell Tissue Res. 2024 Dec 20;399(2):193–209. doi: 10.1007/s00441-024-03938-y (PMC11787269; doi:10.1007/s00441-024-03938-y)
Supplement: Supplementary file 4 — Supplementary file4 (PDF 596 KB) [file 441_2024_3938_MOESM4_ESM.pdf]

Supplementary Figures

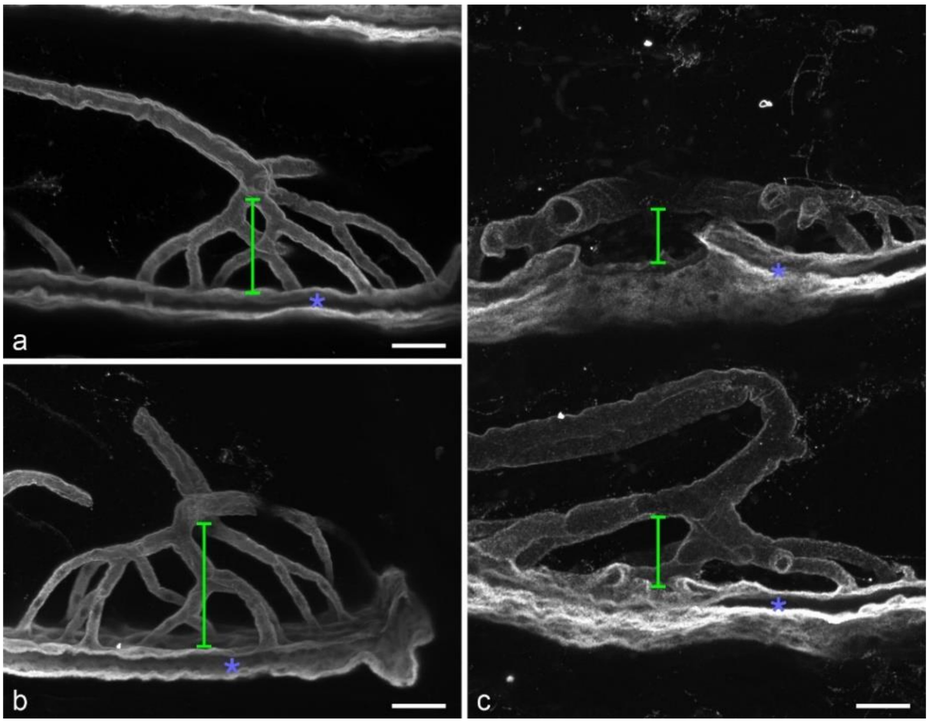

Fig. S1 The stalklet system as viewed from the side. Maximum intensity projections of image stacks taken on vibratome sections that were stained for Na^+/K^+-ATPase α-subunit. Stack thickness is 29 µm (**a**), 51 µm (**b**), 31 µm (**c**). Note that the attachment site of stalklets to the terminal stalk is close to but at keeps a variable distance (green bar) from the electrocyte´s posterior surface. Blue asterisks, main body of the electrocyte. Scale bars: 25 µm

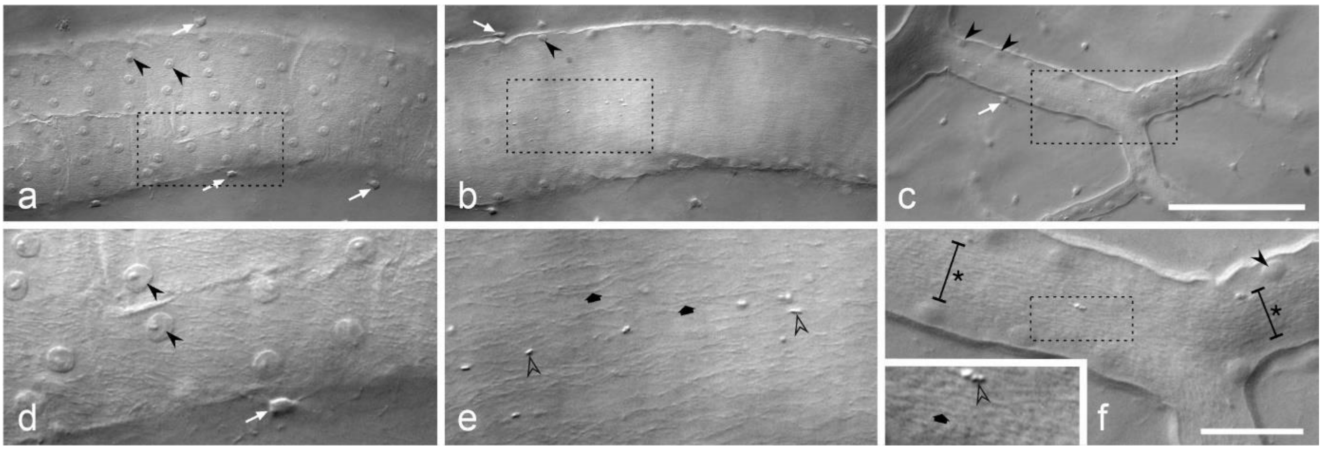

Fig. S2 Fine structure of stalks. Transverse vibratome sections through EOs were imaged by DIC microscopy. **a** Top view on a ~100 µm thick main stalk. **b** Median optical section through the stalk shown in **a**. **c** Optical section of a thinner (Ø ~30 µm) stalk. Areas outlined by dashed lines in **a, b, c, f** are shown at higher magnification in **d, e, f** and **f inset**. Nuclei (black arrowheads) have a peripheral position and a quite uniform distribution in stalks. The centre of stalks (**e**, asterisks in **f**) contains fibrous structures (broad arrows) and oval organelles (open arrowheads). Cells on the stalk surface (black arrows) likely represent fibrocytes. Scale bars: 100 µm (**a-c**), 25 µm (**d-f**)

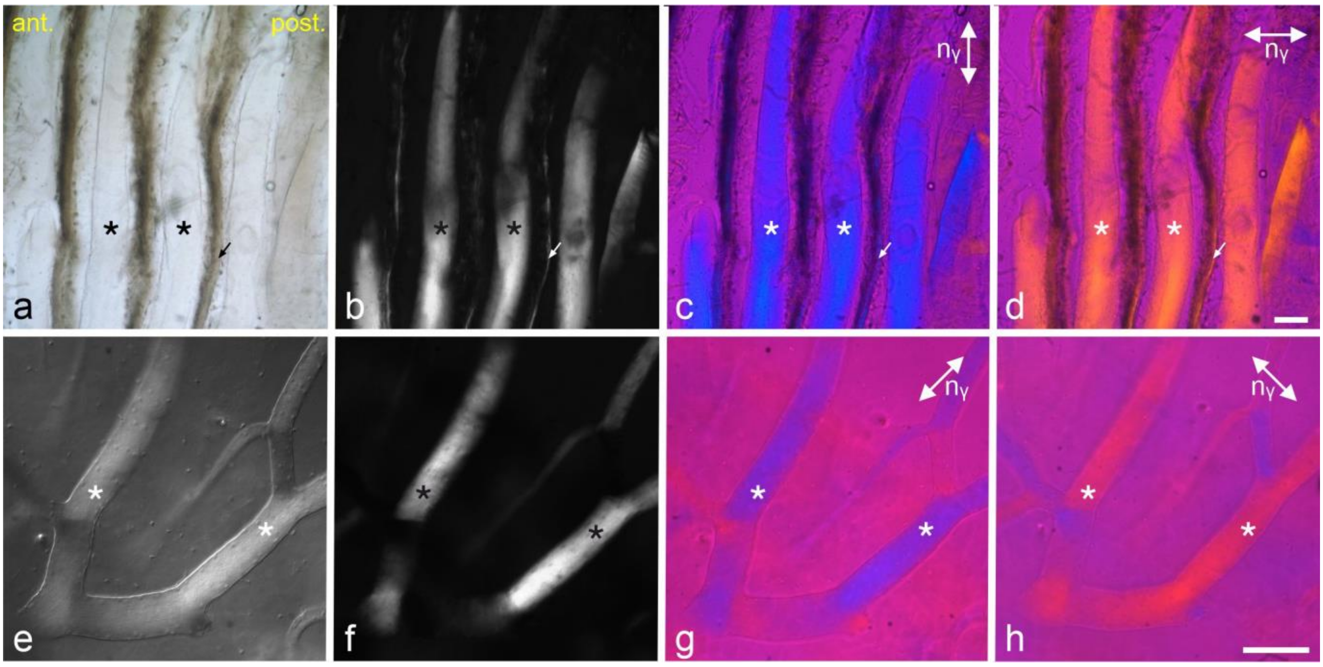

Fig. S3 Stalks are birefringent. **a-d** Main stalks in a sagittal section through an EO; **e-h** thick stalks in a transverse section through an EO. Stalks are presented by brightfield microscopy (**a**), DIC microscopy (**e**) and polarization microscopy without (**b, f**) and with a first order retardation plate (**c, d, g, h**), with the orientation of n_γ rotated by 90 ° (n_γ indicated on the upper right). Interference colours in **c, d, g, h** demonstrate positive birefringence of stalks (asterisks) and of the electrocyte main body (arrows in **a-d**). Scale bars: 100 µm

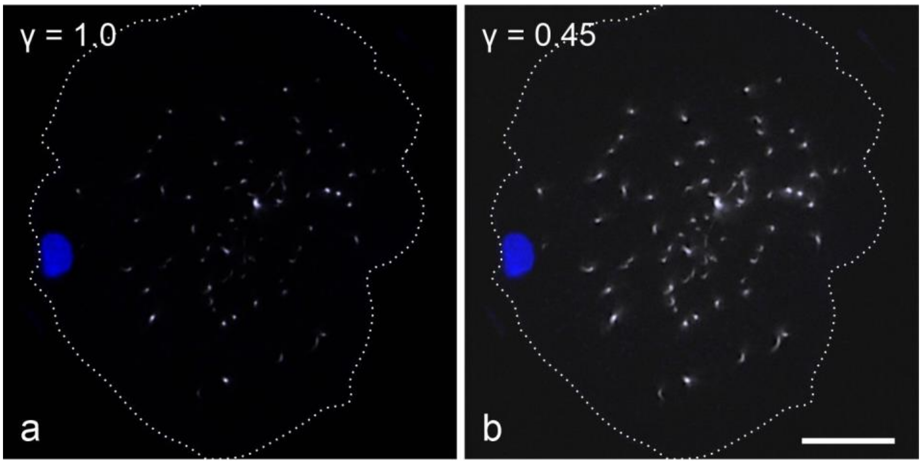

Fig. S4 The F-actin system in stalks. Cross-section through a relatively thick stalk, stained with CF488-phalloidin (white) and DAPI (blue). The plasma membrane is indicated by a dashed line. **a** No gamma correction to visualize the relative fluorescence intensity of F-actin structures. **b** Gamma correction set to 0.45 to highlight the number and position of all F-actin structures. Note that the F-actin structures differ widely in intensity, indicating that they are composed of different numbers of actin filaments. Scale bar: 10 µm
